# The Effect of Maternal Employment on the Elementary and Junior High School Students’ Mental Health in Maku

**DOI:** 10.5539/gjhs.v7n2p379

**Published:** 2015-02-24

**Authors:** Fatemeh Bakhtari Aghdam, Sakineh Ahmadzadeh, Zahra Hassanalizadeh, Fatemeh Ebrahimi, Leila Sabzmakan, Zeinab Javadivala

**Affiliations:** 1 School of Health, Tabriz University of medical sciences, Tabriz, Iran; 2 Health center of Maku, Urmia University of medical sciences, Urmia, Iran; 3 Health center of Bukan, Urmia University of medical sciences, Urmia, Iran; 4 School of Health, Alborz University of medical sciences, Karaj, Iran

**Keywords:** elementary and junior high school, mental health, maternal employment

## Abstract

**Background and Objective:**

Most experts view the childhood period as a foundation for shaping the individuals’ fundamental future characteristics and behaviors. They believe that parents’ personality and behavior quality exert a greater effect on the development of a child’s personality than other factors. Given the mothers’ role in children’s mental health and considering the fact that children are a nation’s future makers, the present study was designed to investigate the impact of maternal employment on students’ mental health in Maku.

**Materials and Methods:**

The present study is descriptive and cross-sectional, and the population of the study encompasses all students in the fifth, sixth, and seventh grades (n=583) who are studying in 2013-2014 academic year in Maku. General Heath Questionnaire was employed for gathering data, and the SPSS software was used for analyzing the data.

**Findings:**

The results of the study indicated that there was a significant difference between the mental health problems, somatic problems, social functioning, anxiety, and depression of the students with employed and non-employed mothers. In other words, the students with non-working mothers experienced greater mental disorders than those with working mothers.

**Conclusion:**

According to the findings of this study, it can be concluded that children with working mothers showed a better mental health than non-working mothers’ children.

## 1. Introduction

The attitude towards children and their nurturing has undergone many ups and downs during the last centuries. Most of the experts consider childhood, especially from the birth through the ages of 5 and 6, as the period for shaping a child’s personality and as a foundation for his/her forthcoming behaviors. They believe that the parental personality, their behavior and the parents are the first factors who play a role in the formation of a child’s sensitive mind (Hosseini Nasab & Khezerluye Aghdam, 2009). Family is one of the most basic and fundamental social institutions in which the child comes into the existence, is raised, and prepares himself/herself for participating in the society and beginning his/her social life. Meanwhile, as one of the two pillars of a family a woman plays dual traditional roles of both mother and wife, which considering her physical and mental characteristics impose some burden of responsibility on her. Managing the house affairs and the family, taking care and bringing up the children, and establishing a friendly emotional atmosphere within the family are among women’s responsibilities. Moreover, in cases which other conditions and facilities are provided, she can bring up healthy and successful children, and accordingly ensure the society’s well-being (Fadayi, 2009).

One area of social life change in the modern age is changes and variations in women’s life. These variations, at large scales, brought about changes in the beliefs and social attitudes towards women as well as changes in the women’s individual, family, and social roles. Among these changes, the women’s employment is of significant importance. The greater effect of employment can be found in the relationship between mothers and their children. The long distance between the work place and the house in official occupations and regular attendance in the work place for long hours are among the factors which attract psychologists’ and experts’ attention. According to some educational psychologists, there is an indispensable relationship between the child’s emotional development and the mother’s presence in the family, especially during the first three years of life. The child’s mental and emotional health depends on the mother’s physical presence at home (Za’aferanchi, 2009).

According to the above-mentioned discussion, the researchers are seeking to find out the effect of mothers’ employment on children’s mental health. A study conducted in this area suggested that there was a significant association between the mothers’ working shift or the length of her presence at home and the children’s social growth (Fadayi, 2009). Other studies revealed that the employment of the mothers of fourth and fifth grade primary students led to social adjustment and higher educational achievement. The children of the working mothers with low social service jobs have shown the least adjustment, and the children of the mothers with cultural and administrative professions reflected the best educational and behavioral performance (Ahmadi & Taghavi, 2003).

In fact, in addition to meeting children’s material needs, a mother’s responsibility is to provide their mental health. Attention deficit and child’s separation from mother can lead to different types of disorders and illnesses including anxiety, fear, depression, and insomnia in the child. Attention deficit during the first four years of life provides a suitable ground for creating anxiety and distress in the child, and in these circumstances, the child manifests aggression and violence which can cause more intensive problems in adolescence (Mirzabeigi & Mirzabeigi, 2009).

Taken the mothers’ significant role in the children’s mental health and considering that children are a nation’s future makers whose well-being significantly affects a society’s health in the years to come, the present study embarked on examining the effect of the maternal employment on the children’s mental health. In so doing, the impact of the mothers’ employment on the students’ mental health is evaluated within the society and special culture of West Azerbaijan, Iran, especially in the city of Maku. This study examined the effect of maternal employment in a sample of elementary and junior high school students’ mental health.

## 2. Material and Methods

The present study was descriptive cross-sectional and was carried out on 583 fifth, sixth, and seventh grade students studying in 2013-2014 academic year in Maku, West Azerbaijan, Iran. After referring to the general education office and doing the necessary arrangements with Education staff, eight schools out of 30 were selected through quota sampling from different parts of the city across different socio- economical classes. Out of the eight schools, three were junior high schools and five were elementary schools. Then 583 students among all students in the fifth, sixth, and seventh grades were included in the study. The criteria for inclusion were that all students selected for the purpose of study were studying and willing to participate. The criteria for exclusion were continual absences, unwillingness to participate in the study, and single-parent students. The data collection instrument utilized in this study was 28-item General Health Questionnaire (GHQ-28). This self-administration questionnaire included 28 items developed by Goldberg and Hillier (1979) divided into four subscales, and each scale comprises of seven items regarding somatic symptoms, anxiety, social functioning, and depression symptoms. Items 1 through 7 were related to the somatic symptoms scale, items 8 through 14 were devoted to anxiety scale, items 15 to 21 dealt with social functioning scale, and questions 22 to 28 were allotted to depression scale (Yaghubi, 2008). In the present study, the students’ mental health scores were measured through GHQ-28 which included four scales as (1-2-3-4). In other words, for options A, B, C, and D the scores were respectively 1, 2, 3, and 4. Therefore, the lowest possible score was 28, and the highest possible score was 112. This questionnaire has been employed in many studies throughout Iran in Persian language, and has indicated high reliability and validity (Balahang H, et al, 2006; Sepehrmanesh, 2008). Because of Persian language is employed in education system in Iran rather than native language, in the present study was used Persian language to data collection. In the present study, GHQ-28 has been shown to have acceptable test-retest reliability (r=.88). The total cronbach alpha for the GHQ-28 in the current sample was .84. The data were analyzed using SPSS version 18. The quantitative variables were reported through means and standard deviation, and the qualitative data were reported using frequency measures. Moreover, the *t*-test was applied to compare two groups.

## 3. Results

The results of the present study showed that 16.6 % of the mothers were employed and the rest were unemployed. The other demographic characteristics are presented in Table 1.

**Table 1:** Characteristics of the Participants based on Gender, Grade and The Mother’s Employment

Variable	n (%)
**Mother’s Employment**	
Unemployed	486 (83.4)
Employed	97 (16.6)
**student’s Grade**	
Grade7	196 (33.6)
Grade6	224 (38.4)
Grade5	163 (28)
**Gender**	
Female	258 (44.3)
Male	325 (55.7)

Data are reported as n (%).

The results of the present study showed that the mental health problems of the unemployed mother’s children are significantly greater than those of the employed mothers. Moreover, the rate of depression and anxiety among the students with employed mothers was significantly lower than that of the children with unemployed mothers. Somatic problems and social functioning of the students with unemployed mothers was higher than those of the students with employed mothers, which was statistically significant. The results of *t*-test in the present study revealed that the students with working mothers were in better conditions in terms of mental health, depression, anxiety, somatic problems, and social functioning compared to the students with nonworking mothers (Table 2).

**Table 2:** Comparison of mental health, anxiety, depression, somatic problems, and social functioning between students with and without employment mothers

Employed mother	Unemployed mother	Variable*
	Mean(SD)	Mean(SD)
Mental Health^‡^	40.2(9.6)	43.9(9.0)
Anxiety^‡^	13.8(3.8)	12.5(3.2)
Depression^‡^	5.4(1.8)	5.0(1.9)
Somatic problems^‡^	12.5(1.2)	11.2(1.2)
Social functioning^‡^	12.1(3.09)	11.4(2.7)

Data reported as mean (Standard Deviation).

‡ P>0.05.

Since mental health questionnaire items test mental health problems practically, and are designed in a way that they measure anxiety level, somatic problems, social dysfunction, and depression in case of respondent’s agreement. On the other hand, mental health construction is exactly in contrast with these cases. It should, therefore, be noted that lower scores indicate higher health, less anxiety, less depression, somatic problems, and social dysfunction.

## 4. Discussion

The results of this study indicated that, contrary to expectations, the mental health problems of the working mothers’ students were lower than those of non-working mothers in four components of anxiety, depression, mental health, and social functioning. The reason may lie in the fact that the working mothers had at least a bachelors degree, and higher education can have considerable effect on their mental health and patterns of communication with their children so that it can increase the mental health of the students of working mothers as compared to that of the students with non-working mothers. Cording to other study it is possible that mothers’ education moderates the link between their work and parenting quality so their children experience lower mental health problems. Augustine (2014) showed that nonemployment among less educated women was associated with the lowest levels of parenting quality (Augustine JM, 2012).^1^

Another factor that can influence in this relationship is mother’s number of working hours. Students whose mothers were employed part time exhibited an advantage in academic learning because of increased rates of school participation and parent-child interaction, whereas students of mothers employed full time appeared to experience a lower learning growth, given lower rate of school participation and fewer educational trips than students of unemployed mothers. (Youn MJ, et al, 2012, Crosnoe R, et al., 2012). Another astudu indicated that whereas early maternal employment does not have an effect on a child’s academic development, recent maternal employment (during a child’s adolescent years) significantly decreases grades (Baum CL, 2004). However, a meta-analysis of 68 studies (770 effect sizes) used random effects models to examine whether children’s achievement differed depending on whether their mothers were employed, showed that When all employment was compared with nonemployment for combined and separate achievement outcomes without moderators, effects were nonsignificant (Goldberg WA, et al, 2008). Therefore, further research should be conducted by including more variables (e.g., job satisfaction, doing work since the child’s birth or even prior to that or after the early years of the child’s birth, the degree of family support, especially the spouses of the working mothers, and so on). In other words, we need further variables to more accurately judge the mental health level of students with working and nonworking mothers.

The analysis of the means showed that in the present study the children with non-working mothers have more problems than the children with working mothers which is in line with the results of some other studies. The results of a study by CusWorth (2006) revealed that the parental employment and non-employment and the children’s mental and educational health will affect the children in different ways, through diverse mechanisms, and with their own special outcomes including the effect on the family income level and the amount of time parents spend with their children. For example, the students living in low-income families were more subject to the problems resulting from lack of education and discipline. In addition, the mothers with part time jobs could ensure their children’s mental health.

The second finding of the present study indicated that the anxiety scores of the students with working mothers were lower than those of the non-working mothers, revealing that this finding is in line with findings of some other studies and in contrast with the findings of some others.

A study by Abster and Khanjani (2009) on 12-14 year-old junior high school students showed that there was a significant positive correlation between the mother’s employment and children’s hyperactivity, stubbornness, disobedience, psychosis, and developmental learning. In other words, these disorders were less manifested in children with housewife mothers than children with working mothers, which does not support the results of the present study. However, the results of some studies indicated that the children with parents diagnosed with anxiety disorder (resulting from factors such as unemployment, poverty, and cultural deprivation) manifested more anxiety disorders (Beidel & Turner, 1997; Biederman et al., 1991; Merikangas et al., 1998). However, it is noteworthy to mention that, in these studies, other research instruments such as interview and observation were employed which can exert different influence on the results.

Furthermore, the results of the present study revealed that there was a significant relationship between the depression scores of the students with working and nonworking mothers, which confirms the results of some studies and disconfirms some others. According to Spitz, the stage in which the mothers shape their love for children on which child’s emotions are founded is a critical period. Partial or complete lack of mother’s presence in a child’s first year of life can contribute to different degrees of anxiety, depression, and lack of readiness to subsequent life problems (Dadsetan, 2007). A study on 40 elementary school children with working mothers showed that they had problems in their relationship with other people, and some symptoms of isolation, withdrawal, and lack of self-confidence were observed. Besides, the emotional symptoms were more evident in children whose parents left them, or those who spent most of their time outside (Za’aferanchi, 2009). The research indicated that the mothers’ negligence in the early years of the child’s life due to employment, and leaving the children in the kindergartens had negative effects on the male children, while the female children with working mothers showed greater level of learning, academic achievement, and self-confidence as compared to those with nonworking mothers, and they were even more successful in pursuing different professions (Fadayi, 2009). It should be mentioned that the age of samples in other studies was lower than the current study, and in the present study it was unknown whether the mothers were employed since the birth or they became employed later on. Additionally, a study by Baum (2003) demonstrated that mother’s income decreased the negative effects of the mother’s early employment on the awareness or cognitive outcomes, and the added income resulting from mother’s employment raised financial security. It also helped ensuring the fulfillment of the children’s primary needs like food and clothing, increased the financial resources and children’s learning opportunities, and reduced family stress, all of which had positive effects on the child’s achievement (Goldberg, Prause, & Lucas-Thompson, 2008).

The present study revealed that the mean of somatic problems for children with working mothers was lower than that of the students with nonworking mothers. This finding is congruent with some studies. In a study by (Cote et al., 2008) it was found that the children who attended kindergarten and were looked after by people other than their mothers, revealed greater aggressive and behavioral problems than the children not attending kindergarten. This issue was only true with regard to the lower classes of the society; that is, in the middle and high social classes no significant difference was observed among the children (Abster & Khanjani, 2009). In addition, a study by Hushang (2010) showed that the mothers’ employment had positive effects on reduced rate of emotional-behavioral disorders among the children. In the same vein, the results of Baum’s (2003) study also verified the results of the present study.

Moreover, the results of the present study denoted that there was a significant relationship between the social functioning of students with working and nonworking mothers, which is in line with the results of some studies and different from results of some others. A study by Ahmadi and Taghavi (2003) suggested that the children of the mothers working in low service jobs showed the least adaptation, and the children who had mothers with cultural and managerial jobs showed the best academic and behavioral function. However, Hosseini Nasab and Khezerlu (2009) found that the rate of adaptation among the female students with housewife mothers was higher than rate of the employed mothers’ children. On the other hand, the female children of working mothers interested in their jobs manifested the highest adaptation mean, while adaptation mean of female students whose mothers worked due to financial needs and with the purpose of independence was the lowest. Additionally, the results of study conducted by Hoffman (1998) showed that the female children with working mothers were more independent and obtained higher scores especially in interaction with their peers at school, in social activities, and in regulating social emotions. Moreover, the female children of working mothers had more self-confidence among different groups, were less shy, and felt higher sense of efficiency, experienced greater vocational achievement, and were more committed to the work. Furthermore, the working-class male children showed positive social adaptation. The results of studies on male children from different social classes demonstrated that working-class children of employed mothers showed satisfying academic achievement as well as positive social adaptation.

Employment affects the women’s personality and awareness which subsequently impacts the family members. If mothers are satisfied with their lives and feel comfortable, they can create positive mental and psychological characteristics in their children. The emotional growth and mental health of a child depends on home and family environment. The homeless children facing the parents’ autocracy and discrimination rather than their affection and love will tend to be sensitive, touchy, and maladjusted people. Therefore, parents are responsible for their children, and they should be prepared for their responsibilities. All in all, the present study indicated that the mental health problems of non-working mothers’ children were higher than those of the students with working mothers, and this difference was significant. Given that the data collection instrument in the present study was a questionnaire, and such data collection techniques such as interview with the parents, observation, and interview with the children themselves were not applied, the results of this study are only valid at this level. Moreover, since the participants were between ages 11 through 13, we cannot generalize the findings to other groups and populations. The results of the present study showed that the mothers’ employment had positive effects on older children. In the present study, the mothers with six or more than six-working hours were considered as employed. Different studies indicated that children under the age of three should be taken care at home by their mother or a nurse, but after that, since their social character is being formed, they need to interact with other children. The main limitation of the present study is contributed to the fact that it lacked any information related to the past and the first three years of the participants’ life, and the judgments can only be made based on the results. Other limitation was the fact that students in Azarbayjan are usually speaking in Turkish language rather than national language which is employed in education system had not been taken into account in this study.

## 5. Conclusion

According to the results of the present study, it can be concluded that the children of working mothers had better mental health than the children with nonworking mothers. However, this study still suffers from some limitations which may affect the results including determining the exact effect of the cultural and social factors besides other factors, the type of mother’s job, working hours, segregation since birth or after the age of 3 from the mother, the socio-economical class, the degree of family support and companionship, especially support from working women’s spouse, and their cooperation in bringing up the children and managing the house affairs.
